# Increased hsa-miR-100-5p Expression Improves Hepatocellular Carcinoma Prognosis in the Asian Population with *PLK1* Variant rs27770A>G

**DOI:** 10.3390/cancers16010129

**Published:** 2023-12-27

**Authors:** Zhouxiang Liao, Qi Zhang, Lichao Yang, Hui Li, Wanling Mo, Zhenyu Song, Xuejing Huang, Sha Wen, Xiaojing Cheng, Min He

**Affiliations:** 1 School of Public Health, Guangxi Medical University, Nanning 530021, China; liaozhouxiang@stu.gxmu.edu.cn (Z.L.); lihui@stu.gxmu.edu.cn (H.L.); 202020957@sr.gxmu.edu.cn (W.M.); songzhenyu@sr.gxmu.edu.cn (Z.S.); chengxiaojing@gxmu.edu.cn (X.C.); 2 Laboratory Animal Center, Guangxi Medical University, Nanning 530021, China; 202120004@sr.gxmu.edu.cn (Q.Z.); yanglichao@gxmu.edu.cn (L.Y.); huangxuejing@sr.gxmu.edu.cn (X.H.);; 3 Life Sciences Institute, Guangxi Medical University, Nanning 530021, China; 4 Key Laboratory of High-Incidence-Tumor Prevention & Treatment (Guangxi Medical University), Ministry of Education, Nanning 530021, China

**Keywords:** hepatocellular carcinoma, miRNAs, prognosis, hsa-miR-100-5p, *PLK1*, rs27770

## Abstract

**Simple Summary:**

Hepatocellular carcinoma (HCC) has the highest incidence and mortality rate in the Asian population. However, it is unclear how ethnic genetic variability affects this. Our research shows that hsa-miR-100-5p, a miRNA associated with HCC prognosis, improves prognosis in Asians. In contrast, Polo-like kinase 1 (*PLK1*), inhibited by hsa-miR-100-5p, worsens HCC prognosis in Asians. A single-nucleotide polymorphism (SNP) named rs27770, located in the 3′UTR of PLK1, has a significantly higher frequency of the G allele in the East Asian population. The A>G change in rs27770 affects the *PLK1* mRNA secondary structure and alters the hsa-miR-100-5p/PLK1 interaction by creating an additional seedless binding site. This could make hsa-miR-100-5p more favorable for HCC prognosis in the Asian population. Our findings could help explain why the Asian population is more susceptible to HCC.

**Abstract:**

Hepatocellular carcinoma (HCC) has the highest incidence and mortality in the Asian population, and race is an independent risk factor affecting survival time in liver cancer. Micro RNAs (miRNAs) are remarkably dysregulated in HCC and closely associated with HCC prognosis. Recent studies show that genetic variability between ethnic groups may result in differences in the specificity of HCC miRNA biomarkers. Here, we reveal a high expression level of hsa-miR-100-5p, an HCC prognosis-related miRNA, which improves HCC prognosis in the Asian Population with Polo-like kinase 1 (PLK1) variant rs27770A>G. In this study, we discovered that hsa-miR-100-5p was downregulated in various HCC cell lines. While mimics transient transfection and mouse liver cancer model confirmed the interaction between hsa-miR-100-5p and *PLK1*, a stratified analysis based on the Cancer Genome Atlas Liver Hepatocellular Carcinoma (TCGA-LIHC) data suggest both low hsa-miR-100-5p expression level and high *PLK1* expression level associated with poor HCC prognosis, especially in the Asian population. According to the 1000 Genomes Project database, the SNP rs27770 located in 3′UTR of *PLK1* had a significantly higher G allele frequency in the East Asian population. Bioinformatics analysis suggested that rs27770 A>G affects *PLK1* mRNA secondary structure and alters the hsa-miR-100-5p/*PLK1* interaction by forming an additional seedless binding site. This racial variation caused *PLK1* to be more vulnerable to hsa-miR-100-5p inhibition, resulting in hsa-miR-100-5p being more favorable for HCC prognosis in the Asian population.

## 1. Introduction

Liver cancer is the sixth most diagnosed cancer and the fourth leading cause of cancer-related deaths worldwide, with the highest incidence and mortality in East Asia and Africa [1]. Hepatocellular carcinoma (HCC) is the most commonly diagnosed liver cancer, and its main risks include cirrhosis, alcohol consumption, aflatoxin exposure, hepatitis B virus and hepatitis C virus infection, and non-alcoholic fatty liver disease [2]. While most cases of HCC are reported in East and Southeast Asia, evidence shows racial variation is a factor in HCC susceptibility in the Asian population [3]. Studies in the United States reveal that Asian Americans show the second highest incidence but lowest mortality rates of liver cancer, indicating that incidence rates and HCC prognosis differ with race and region [4].

MicroRNAs (miRNAs) are small endogenous single-stranded non-coding RNAs that have been recognized as playing essential roles in cancer prognosis by regulating a wide range of biological processes, typically via sequence-specific RNA-RNA interactions [5,6]. Due to various mutations, miRNAs are heavily dysregulated in cancer cells, leading to the dysregulation of multiple protein-coding oncogenes [6,7,8,9,10]. miRNAs are crucial regulators in the development and progression of HCC [11,12,13]. miRNA dysregulation in HCC leads to the deregulation of cell death, cell cycle, or other key signaling pathways, resulting in the uncontrolled progression of HCC cells [12]. hsa-miR-100-5p is a widely known tumor suppressor that regulates various cell processes such as cell proliferation, differentiation, migration, invasion, and apoptosis by post-transcriptionally regulating several oncogenes [14,15]. The downregulation of hsa-miR-100-5p in HCC correlates with tumor progression and poor prognosis [16,17,18,19]. One of the target genes of mir-100 is Polo-Like Kinase 1 (*PLK1*), a proto-oncogene that encodes a serine/threonine-protein kinase that plays a crucial role in cell cycle regulation and is a potential therapeutic target for HCC [19,20]. Recent studies have shown that Asian-specific HCC miRNA biomarkers are far more than non-Asian ones, suggesting that genetic variability between ethnic groups may result in the ethnic specificity of HCC miRNA biomarkers [21].

Single-nucleotide polymorphisms (SNPs) are inherited mutations distributed throughout the genome and may act as biomarkers to reflect the genetic differences among races, populations, and individuals [22]. Genome-wide association studies (GWAS) involve large-scale cancer-related SNP analysis to identify numerous possible genetic loci associated with features of heterogeneous diseases, such as cancer susceptibility, survival prognosis, and drug response [23,24,25]. However, the mechanisms by which these SNPs affect cancer risk remain largely unknown. Approximately 11% of all SNPs are located in the 3′-UTR of genes, potentially disturbing their binding by microRNAs [26,27,28]. SNPs located in mature miRNA coding regions or target sites could disturb miRNA-mRNA interactions, altering miRNA-regulated gene expression, which is implicated in various human diseases, including cancer [26,29,30].

In this study, we found that the downregulation of has-miR-100-5p has an exceptionally high impact on the prognosis of HCC in the East Asian population. In addition, we investigated the has-miR-100-5p target gene *PLK1*. We discovered an SNP named rs27770A>G, which is located in 3′UTR and has a higher allele frequency in the East Asian population, which alters the *PLK1* mRNA stability and potentially enhances the has-miR-100-5p/*PLK1* interaction.

## 2. Materials and Methods

### 2.1. Cell Lines and Cell Culture

Normal human hepatic cell line HL7702 and hepatocellular carcinoma cell lines Huh7, HepG2, and Hep3B were bought from the China Center for Type Culture Collection. HL7702, Huh7, and HepG2 were cultured in the DMEM high glucose medium (Cat 11965092, GIBCO, Grands Islands, NY, USA) supplied with 10% FBS (Cat 10099158, GIBCO, Grands Islands, NY, USA). Hep3B was cultured in the MEM medium (Cat 11095080, GIBCO, Grands Islands, NY, USA) with 10% FBS. All cells were cultured at 37 °C with 5% CO_2_.

### 2.2. RNA Isolation, Library Construction, and Sequencing

Total RNA from the four cell lines was extracted using TRIzol reagent (Cat 15596026, Invitrogen, Waltham, MA USA). The quality of total RNA was analyzed using the Bioanalyzer 2100 system (Agilent, Santa Clara, CA, USA). sRNA libraries were prepared from total RNA for the DNBSEQ-G400 platform (MGI Tech, Shenzhen, China) using MGIEasy Small RNA Library Prep Kit (Cat 940-000196-00, MGI Tech, Shenzhen, China). Sequencing was performed on DNBSEQ-G400 until each library yielded more than 25 million raw tags, with a read length of 50. All low-quality, invalid, polyA-containing, and short tags were removed, resulting in clean tags aligned with the GRCh38.p12 reference genome using Bowtie2 and processed with UMI_tools to reduce the quantitative bias introduced by PCR. The accurate quantity of sRNAs was then calculated.

### 2.3. Data Acquisition and Statistic Analysis

All statistical analyses were conducted using R software version 4.0.3, IBM SPSS Statistics 25.0 software, and GraphPad Prism 9.4.0 software. Clinical data from the TCAG-LIHC project were downloaded from the TCGA Research Network (https://www.cancer.gov/tcga (accessed on 25 May 2023)). The expression level and survival data of hsa-miR-100-5p and *PLK1* were downloaded from OncoLnc (http://www.oncolnc.org (accessed on 25 May 2023)) [31]. Survival analyses were performed to determine miRNA and mRNA dysregulation hazard ratio. For each RNA of interest, the best cut-points of expression level were determined by X-tile software (version 3.6) [32], and then all survival analyses were performed using SPSS software. A separate Log-rank test of equality of all factor levels was performed for each stratum. Experimentally validated miRNA-mRNA interaction data were downloaded from miRTarBase (https://mirtarbase.cuhk.edu.cn/~miRTarBase/miRTarBase_2022 (accessed on 6 June 2023)) [33]. Datasets containing SNP allele frequency and genotype frequency of genes of interest identified by 1000 Genomes Project Phase 3 [34] were exported from Ensembl by BioMart (https://www.ensembl.org (accessed on 14 July 2023)). The equality of all major population subgroups was tested using a chi-squared test to determine whether the allele frequency and genotype frequency significantly differ from the global population and whether or not these genotype distributions fit the Hardy–Weinberg equilibrium.

### 2.4. q-PCR Analysis Expression Level of miRNAs and mRNAs

All primers used for q-PCR were listed in Supplementary Table S1. To analyze the expression level of hsa-miR-100-5p, total RNA extracted from cell lines culture was reverse transcripted into cDNA using miRNA 1st Strand cDNA Synthesis Kit (MR101, Vazyme, Nanjing, China) using stem-loop primer mir-100-5p RT and U6_snRNA_R. Quantitative PCR (q-PCR) was performed on LightCycler 480 (Roche, Basel, Switzerland) using ChamQ SYBR q-PCR Master Mix (Q321, Vazyme, Nanjing, China) using mir-100-5p_qRT_F and StemLoop_R, along with U6_snRNA_F and U6_snRNA_R to amplify U6 snRNA as an internal reference. To analyze the expression level of *PLK1*, total RNA extracted from cell lines culture was reverse transcripted into cDNA using HiScript III RT SuperMix for q-PCR (R323, Vazyme, Nanjing, China). q-PCR was then performed using PLK1_F and PLK1_R (PrimerBank ID 34147632c3) [35], along with GAPDH_F and GAPDH_R as internal reference.

### 2.5. Manipulate miRNA Expression Level by Transient Transfection

To manipulate miRNA levels in cell cultures, hsa-miR-100-5p mimic was synthesized as 5′-AACCCGUAGAUCCGAACUUGUG-3′, along with random control mimic 5′-UUCUCCGAACGUGUCACGUTT-3′ (Gensys Biotech, Nanning, China). The synthetic miRNA mimic was delivered into cells using Lipofectamine™ 3000 Transfection Reagent (Cat L3000008, ThermoFisher Scientific, Waltham, MA, USA) following the manufacturer’s instructions. The total RNA was extracted from the cell culture 48 h after transfection, and then miRNA and target mRNA expression were analyzed via q-PCR.

### 2.6. Western Blot Analysis

Briefly, the adherent cell culture was washed with PBS on ice. Then, whole-cell lysates were prepared via cell lysis using RIPA Lysis Buffer (P0013B, Beyotime Biotech, Shanghai, China) containing 1X Protease and phosphatase inhibitor cocktail (P1045, Beyotime Biotech, Shanghai, China) and subjected to ultrasonication. The total protein concentration in the lysate was determined using the BCA Protein Assay Kit (P0012, Beyotime Biotech, Shanghai, China). Then, 20 μg of proteins was subjected to 10% SDS-PAGE. The separated protein bands were immunoblotted onto polyvinylidene difluoride membranes (ISEQ 000 10, Millipore, Burlington, MA, USA). The membranes were blocked with 5% non-fat milk powder in TBS buffer. Primary antibodies were incubated overnight at 4 °C followed by secondary antibodies for 2 h at room temperature. After adding Super Signal West Pico PLUS substrate (Cat 34580, ThermoFisher Scientific, Waltham, MA USA), chemiluminescence signals on the membrane were visualized using iBright FL1000 (ThermoFisher Scientific, Waltham, MA USA). The antibodies used in this study were anti-PLK1 rabbit polyclonal antibody (1:500 dilute; D321829, Sangon Biotech, Shanghai, China), anti-GAPDH rabbit polyclonal antibody (1:6000 dilute; D110016, Sangon Biotech, Shanghai, China), and HRP-conjugated goat anti-rabbit IgG (1:6000 dilute; D110058, Sangon Biotech, Shanghai, China).

### 2.7. Mice Hydrodynamic Tail Vein Injection (HTVi) Liver Cancer Model

Procedures were as described previously [36,37,38]. Briefly, 12 six-week-old male C57BL/6J mice were randomly divided into two groups (*n* = 6) for HTVi treatment. For the tumor group, a carcinogenic plasmid mixture contains pCMV/SB, pT3-EF1a-C-Met, pT3-N90-β-catenin, lentiCRISPR-Pten, and lentiCRISPR-P53 with1:2:2:2:2 ratio in sterile saline with the final concentration of 5.4 μg/mL was used to achieve Myc^OE^/Ctnnb1^OE^/Pten^KO^/Trp53^KO^ introduced to the liver cancer model. For the control group, an empty vector plasmid mixture containing pCMV/SB, pT3-EF1a, and lentiCRISPRV2 with a 1:4:4 ratio was used instead. All plasmids were prepared with QIAGEN Plasmid Kits (Cat. 12143, QIAGEN, Hilden, Germany) and pre-mixed before HTVi treatment. Before the injection, the mice were weighed. A plasmid mixture of 0.1 mL per gram of body weight was prepared and filled into a 1 mL syringe with a 27G needle for a single dose. The mouse was then restrained, and the tail was cleaned with an alcohol swab. The needle was inserted almost horizontally into either of the two lateral tail veins. The plasmid mixture solution was delivered via rapid injection into the lateral tail vein within 3–5 s. After a recovery period of 30 min, the mouse was returned to its original cage and housed normally. Mice were killed at four weeks after injection. Livers were collected and rinsed in PBS. Part of the sample was preserved for histological analysis. Then, the tumor and normal liver tissues were snap-frozen in liquid nitrogen for RNA extraction. Mice were housed, fed, and monitored by protocols approved by the Laboratory Animal Center of Guangxi Medical University. All experiment protocols were approved by the Animal Care & Welfare Committee of Guangxi Medical University. (No. 202302100)

### 2.8. mRNA Secondary Structure and miRNA Binding Site Prediction

To predict rs27770 A>G effects on local RNA secondary structure, we used RNAsnp (https://rth.dk/resources/rnasnp/ (accessed on 15 July 2023)) mode 1 in the global folding (RNAfold) mode with and the default parameter by setting the folding window to 500. The hsa-miR-100-5p binding site on *PLK1* rs27770–A and rs27770:G were predicted by STarMir (https://sfold.wadsworth.org/cgi-bin/starmirWeb.pl (accessed on 1 August 2023)) using the V-CLIP model. The two results were compared to identify the diverse effects of rs27770.

## 3. Results

### 3.1. sRNA Sequencing Reveals Differential Expression of miRNAs between Normal Liver Cells and HCC Cells

**Figure 1:**
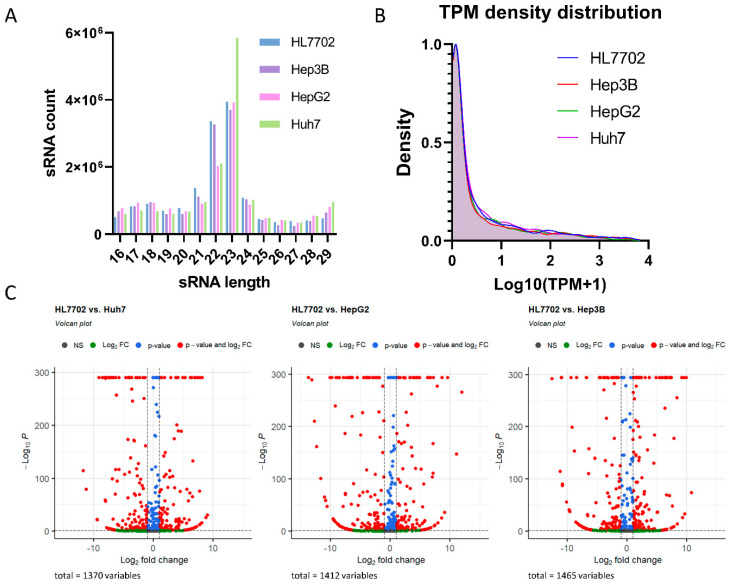
Small RNA sequencing revealed differentially expressed miRNAs in three HCC cell lines compared to normal liver cells. (**A**) sRNA length distribution indicated a normal sRNA length peak range between 21 and 24 nt. (**B**) TPM density distribution for all four cell lines showed similar sRNA abundance tender. (**C**) Volcano plots show the distribution of differentially expressed genes in the three HCC cell lines compared to normal liver cells.

The total RNAs extracted from four different cell lines were built into sRNA libraries and sequenced separately, resulting in about 20 million high-quality clean tags per sample. The sRNA length peak ranged from 20 to 23 nucleotides, and the TPM distribution of the four libraries was very similar to each other (Figure 1A,B), resembling typical sRNA sequencing results. A total of 1655 miRNAs were identified and quantified by mapping the tag alignment using mRBase 22.1 and Rfam12.2, which yielded 1373 known and 282 novel miRNAs.

To identify significant differentially expressed miRNA between normal liver and HCC cell lines, the miRNA expression levels in Huh7, HepG2, and Hep3B were compared with those in HL7702. Then, differentially expressed miRNAs with an absolute value of log_2_(Fold Charge) > 1 and FDR < 0.001 were filtered. Compared to HL7702, 275, 283, and 291, differentially expressed miRNAs were observed in Huh7, HepG2, and Hep3B, respectively (Figure 1C).

### 3.2. hsa-miR-100-5p Is a Tumor-Suppressing miRNA and Is Suppressed in HCC Cell Lines

**Figure 2:**
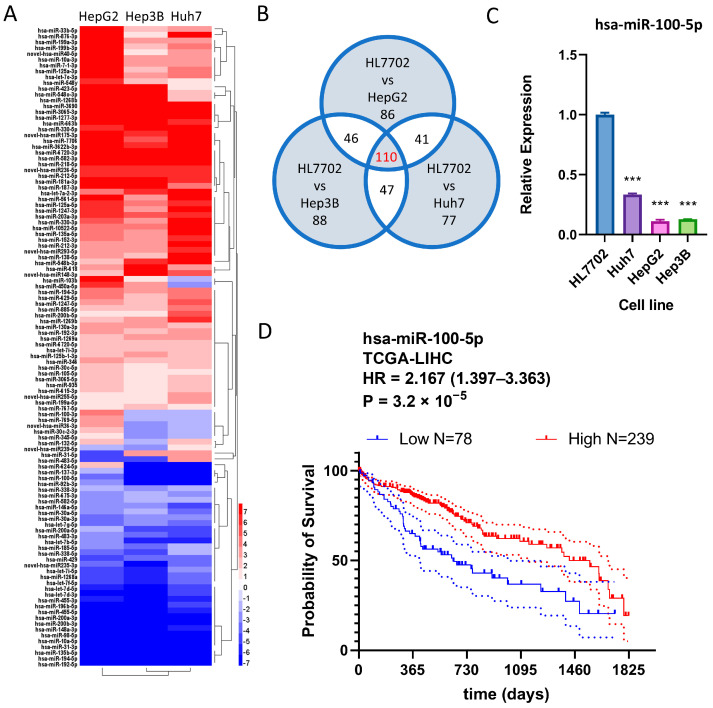
hsa-miR-100-5p was downregulated in all three HCC cell lines and favored overall survival. (**A**) Heatmap showing the 110 commonly differentially expressed genes (DEGs) in Huh7, HepG2, and Hep3B cell lines. (**B**) The Venn diagram shows DEG counts in each set. (**C**) q-PCR showed decreased hsa-miR-100-5p expression in HCC cell lines compared to normal liver cells; *** *p* < 0.001 by student-T tests against miRNA expression levels in HL7702. (**D**) Kaplan–Meier plot based on TCGA-LIHC data shows that increased hsa-miR-100-5p expression is associated with good HCC prognosis. Compared using the Log-rank (Mantel–Cox) test, with follow-up the threshold of 5 years. The dotted lines indicated the 95% CI range.

The dysregulated expression of miRNA genes is a common occurrence in HCC and may influence patient prognosis [11,12,13]. To identify common miRNA changes in HCC, we analyzed Huh7, established from a 57-year-old Japanese male, HepG2, established from a 15-year-old Argentinean Caucasian male, and Hep3B, established from an 8-year-old black male. Of 1655 differentially expressed miRNAs, 110 were consistently differentially expressed in all three HCC cell lines, indicating they were commonly dysregulated in HCC (Figure 2A,B). To determine whether these miRNAs were associated with HCC prognosis, the correlation between the dysregulation of these miRNAs and TCGA-LIHC patient prognosis was investigated using OncoLnc web tools. While both miRNA hsa-miR-769-5p and hsa-miR-1269a, implicated in HCC, were overexpressed in HCC cell lines, hsa-miR-100-5p, which promotes overall survival in HCC patients, was the only HCC prognosis-related miRNA that showed downregulation in three HCC cell lines (Table 1). hsa-miR-100-5p was selected for further investigation due to its highest hazard rating among all candidate dysregulated miRNAs. The downregulation of hsa-miR-100-5p in HCC cell lines was confirmed via q-PCR (Figure 2C). With optimized expression cut-point, survival analysis based on TCGA-LIHC data indicated that low hsa-miR-100-5p expression is associated with significantly worse overall survival time (HR = 2.167, 1.397–3.363 95%CI) (Figure 2D), suggesting that hsa-miR-100-5p is a tumor-suppressing miRNA.

### 3.3. hsa-miR-100-5p Overexpression in HCC Results in PLK1 Downregulation

**Figure 3:**
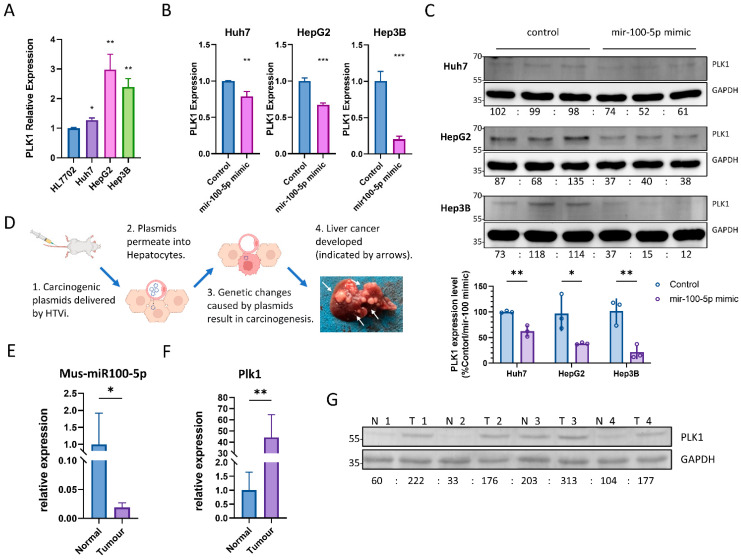
Hsa-miR-100-5p targets well-known oncogenes gene PKL1. (**A**) q-PCR revealed increased PLK1 expression in HCC cell lines compared to normal liver cells; * *p* < 0.05, ** *p* < 0.01 via Student’s *t*-test against mRNA expression level in HL7702. (**B**) Transient transfection of hsa-miR-100-5p mimic inhibited PLK1 expression in HCC cell lines; ** *p* < 0.01, *** *p* < 0.001 via Student’s t-test against control (the same cell line transfected with scrambled miRNA control) PLK1 expression level. (**C**) The results of a Western blot analysis indicate that the transient transfection of hsa-miR-100-5p-mimic led to a reduction in PLK1 protein expression in HCC cell lines. GAPDH was used as an internal control in each cell line, and the average densitometry reading of each cell line was normalized to 100, respectively. The statistical analysis showed that the reduction was significant, with * *p* < 0.05 and ** *p* < 0.01 by Student’s *t*-test compared to the control. (**D**) The diagram demonstrated the hydrodynamic transfection of the liver cancer mouse model. The picture of a mouse liver with tumors was taken four weeks after treatment. (**E**) q-PCR showed decreased mus-miR-100-5p (mouse ortholog of hsa-miR-100-5p) expression in mouse liver tumors compared to normal liver; both the tumor group and normal group represent assay results from 5 individuals; * *p* < 0.05 via Student’s *t*-tests against miRNA expression levels in normal liver. (**F**) q-PCR showed increased *Plk1* (mouse ortholog of *PLK1*) expression in mouse liver tumors compared to the normal liver; both tumor group and normal group represent assay results from 5 individuals; ** *p* < 0.01 by student-T tests against mRNA expression levels in the normal liver; (**G**) Western blot analysis shows higher liver Plk1 level in tumor group, compared to the normal group. N1 to N4 represented four individuals in the normal group, while T1 to T4 represented four individuals in the tumor group. The average PLK1/GAPDH densitometry readings from the normal group were normalized to 100.

The dysregulation of cancer-mediating miRNA expression, in turn, controls the dysregulation of several protein-coding oncogenes [7,8,11]. Using miRTarBase, 258 reported hsa-miR-100-5p mRNA targets were found. We shortlisted six well-studied hsa-miR-100-5p target genes (Table 2). Previous studies have reported that the expression of *PLK1* is negatively correlated with that of hsa-miR-100-5p and is higher in HCC tissues than in non-cancerous liver tissues [19,20]. q-PCR analysis revealed that *PLK1* expression is upregulated in HCC cell lines (Figure 3A). The interaction between hsa-miR-100-5p and *PLK1* mRNA was confirmed via the transient transfection of hsa-miR-100-5p mimic into HCC cells, which remarkably reduced PLK1 expression in both mRNA and protein levels (Figure 3B,C). Supplementary Figure S1 and Table S2 present uncropped blots and densitometry readings.

A hydrodynamic transfection liver cancer mouse model was employed to verify the interaction between hsa-miR-100-5p and *PLK1* further in vivo. Four weeks after HTVi treatment, five of six mice in the tumor group developed multiple tumors on the liver, while no tumor could be found in the control group. (Figure 3D) The histological analysis confirmed the in vivo liver cancer model by identifying typical cytoplasmic basophilic HCC tissues in tumor group samples. The tumors were found to be widely distributed along with blood vessels, and there was no clear boundary between the tumor and non-tumor area (Supplementary Figure S2). As a result, we randomly paired mice in the tumor group with the control group to compare tumor tissue against normal liver tissue. We observed a 98% decrease in mus-miR-100-5p (mouse ortholog of hsa-miR-100-5p) expression level in tumor tissue via q-PCR compared to normal liver tissue from the control group (Figure 3E). Meanwhile, *Plk1* (mouse ortholog of *PLK1*) in tumor tissue was increased on both mRNA and protein levels, strongly suggesting that the loss of inhibition from mus-miR-100-5p could greatly impact *Plk1* expression. (Figure 3F,G).

### 3.4. Low Expression of hsa-miR-100-5p Is Associated with Worse HCC Prognosis, Especially in the Asian Population

**Figure 4:**
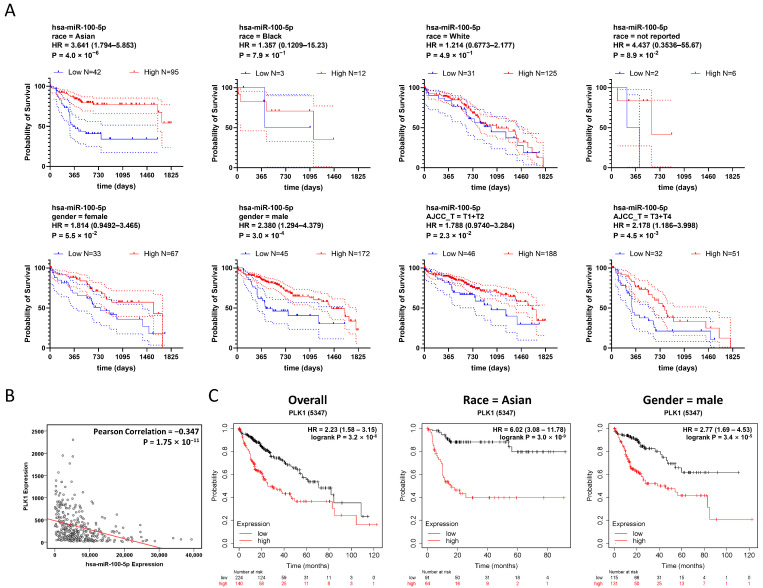
Low expression of hsa-miR-100-5p is associated with worse HCC prognosis, especially in the Asian population. (**A**) Stratified survival analyses demonstrated high hsa-miR-100-5p expression increased the overall survival time of Asian patients with HCC. Kaplan–Meier plot based on TCGA-LIHC data stratified by race, gender, and AJCC staging demonstrating the overall survival of HCC patients with different hsa-miR-100-5p expression levels in various population subgroups. Compared using the Log-rank (Mantel–Cox) test, with follow-up the threshold of 5 years. The dotted lines indicated the 95% CI range. (**B**) *PLK1* expression was negatively correlated with hsa-miR-100-5p. Based on TCGA-LIHC data with the Pearson Correlation model. Each dot represents a case. (**C**) Stratified survival analyses demonstrated that increased *PLK1* expression levels were associated with poor HCC prognosis in the Asian population. Kaplan–Meier plot based on TCGA-LIHC data showing the association of high *PLK1* expression with poor HCC prognosis. Compared using the Log-rank (Mantel–Cox) test.

To investigate the impact of hsa-miR-100-5p dysregulation further, we conducted a stratified survival analysis based on TCGA-LIHC data. TCGA-LIHC cases were stratified by race, gender, and AJCC staging. Overall survival analyses were conducted to analyze survival in low and high hsa-miR-100-5p expression groups (Table 3 and Figure 4A). Of all the ethnic subgroups assessed, Asian patients with low hsa-miR-100-5p expression showed a significantly worse prognosis (hazard ratio = 3.240 (1.794–5.853)), while other ethnic subgroups showed no difference between the low and high hsa-miR-100-5p expression groups (Table 3 and Figure 4A). Meanwhile, gender stratification revealed that men were more susceptible than women to low hsa-miR-100-5p expression (hazard ratio = 2.238 (1.294–4.379)) (Table 3 and Figure 4A). AJCC stage stratification indicated that low hsa-miR-100-5p expression was associated with worse HCC prognosis at all staging, while later stages at which metastasis occurred showed a significantly higher hazard ratio (hazard ratio = 2.178 (1.186–3.998)) compared to early stages. Taking together, stratified survival analysis revealed that hsa-miR-100-5p was a potential Asian specificity prognosis-associated miRNA.

Meanwhile, the correlation analysis based on TCGA-LIHC data revealed a negative correlation between *PLK1* and hsa-miR-100-5p expression (Figure 4B). Survival analysis also showed that increased *PLK1* expression was associated with poor HCC prognosis in TCGA-LIHC patients (HR = 2.23 (1.58–3.15)) (Figure 4C). As *PLK1* was strongly associated with hsa-miR-100-5p, we conducted *PLK1* dysregulation survival analysis for TCGA-LIHC data using the same stratification strategy. The result displayed a correlation pattern from hsa-miR-100-5p, while the Asian subgroup with high *PLK1* expression was associated with the highest hazard ratio (HR = 6.02 (3.08–11.78) (Figure 4C) indicated that *PLK1* dysregulated overexpression may involve the Asian population specificity of hsa-miR-100-5p.

### 3.5. hsa-miR-100-5p/PLK1 Interaction Is Altered in PLK1 rs27770 A>G in the East Asian Population

**Figure 5:**
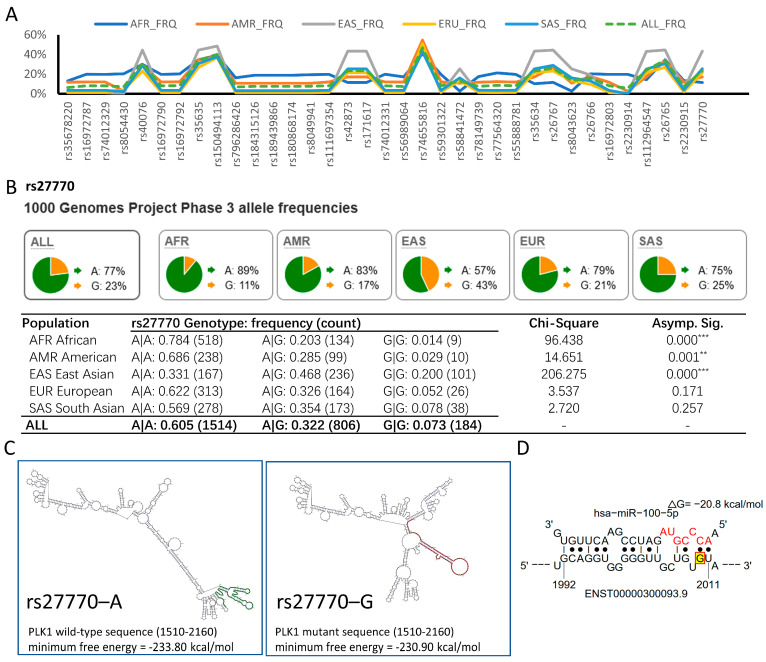
rs27770–G is a high-frequency allele within the East Asian population that could alter the interaction between *PLK1* and hsa-miR-100-5p. (**A**) 1000 Genomes Project Phase 3 data showed that 35 SNPs with a high global MAF have significantly different allele frequencies in the five major population groups. AFR, African; AMR, American; EAS, East Asian; EUR, European; SAS South Asian. (**B**) Allele frequency of rs27770–G was higher in the East Asian population, resulting in significant diversity in the genotypic frequency distribution. The pie chart displays the frequency of alleles for rs27770 in each population, and the table shows genotype frequencies. (** *p* < 0.01, *** *p* < 0.001 via chi-square tests for genotype) (**C**) rs27770–G alters *PLK1* mRNA secondary structure. The major mRNA structural change is marked in red against that of rs27770–A, marked in green. (**D**) rs27770–G resulting in an additional seedless hsa-miR-100-5p binding site between 1992–2011 nt. rs27770–G is highlighted and marked with red basket at 2010 nt.

The genetic basis of the increased susceptibility of Asian patients with HCC to hsa-miR-100-5p and *PLK1* dysregulation was investigated. To identify candidate genetic variants in *PLK1* associated with HCC in the global population, we retrieved 339 SNPs in the *PLK1* coding and untranslated region using the 1000 Genomes Project Phase 3 database. This included 106 common variants with a global minor allele frequency (Global MAF) greater than 0.001. The chi-squared test revealed that of these 106 variants, the allele frequency of 35 SNPs was significantly different in five major population subgroups (Figure 5A).

An important SNP is located in the 3′UTR of *PLK1*, rs27770 2010A>G. Compared with the global population, the allele frequency of rs27770–G was 20% higher in the East Asian population (Figure 5B). In the East Asian population, the genotypic frequency of A|A was 31.1%, A|G was 46.8%, and G|G was 20.0%, which fit the Hardy–Weinberg equilibrium (*p* = 0.571 chi-squared test) (Supplementary Table S2). Moreover, genetic variation data from gnomAD, ExAC, and HapMap also indicated that the allele frequency of rs27770–G is significantly higher in the Asian population (Supplementary Table S3). We also examined the status of rs27770 in the HCC cell lines we use. (Supplementary Data S1). The sequencing results indicated that Huh7 and Hep3B cell lines had the rs27770–A variant, whereas HepG2 had the rs27770–G variant. This suggests that this SNP is quite common across various HCC cell lines.

SNPs can impact mRNA function in various ways, including by disrupting mRNA secondary structures [26,29,30]. We used the RNAsnp webserver to identify *PLK1* mRNA secondary structures potentially disrupted by rs27770–G. The result indicated that the rs27770–G variant significantly alters mRNA secondary structures, resulting in fewer stems and more loops, which may negatively affect mRNA stability (Figure 5C). Using the STarMir webserver, an additional seedless binding site was observed in the rs27770–G variant between 1992 and 2011 nucleotides (Figure 5D) along with 69 hsa-miR-100-5p binding sites, which is the same as the rs27770–A variant (Supplementary Table S4), suggesting that it may be more vulnerable for hsa-miR-100-5p suppression.

**Figure 6:**
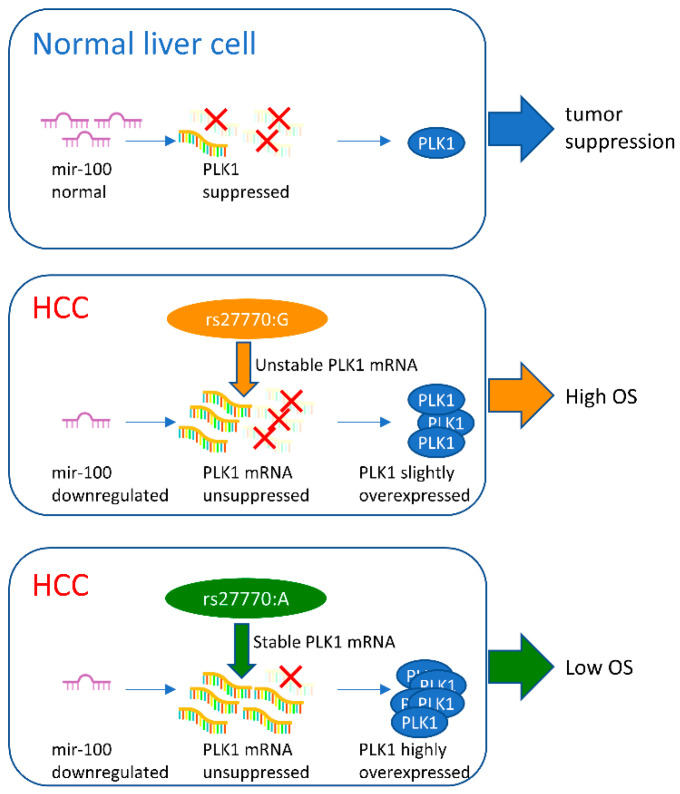
Schematic diagram showing the change in hsa-miR-100-5p/*PLK1* interaction with *PLK1* due to rs27770. Normally, hsa-miR-100-5p expression regulates *PLK1* expression through mRNA degradation (marked with red X). In HCC cells, hsa-miR-100-5p dysregulates downregulation, resulting in disorderly high PLK1 expression associated with bad prognosis for HCC patients. A relatively higher hsa-miR-100-5p expression level combined with the rs27770–G allele in *PKL1* resulted in a less serious *PLK1* disorder related to a better prognosis in Asian HCC patients.

In conclusion, the rs27770–G variant, which is more frequent in the Asian subpopulation compared with the global population, may cause *PLK1* to be more vulnerable to hsa-miR-100-5p inhibition, resulting in hsa-miR-100-5p high expression heavily improving HCC prognosis in the Asian population (Figure 6).

## 4. Discussion

The dysregulation of miRNAs is common in cancers and is caused by various mechanisms, including mutations involving miRNA loci, epigenetic silencing, and transcription factor dysregulation [6,7,8,9,10]. Through sRNA sequencing, we confirmed that hsa-miR-100-5p is downregulated in several HCC cell lines, which suggests a close association between hsa-miR-100-5p dysregulation and HCC. The high abundance of hsa-miR-100-5p in our sequencing results and online database establishes it as an essential miRNA. Previous clinical studies have shown that hsa-miR-100-5p is dysregulated in HCC and other cancers, indicating that its dysregulation is common in cancer [15,16,17,18,19]. While it has been established that the downregulation of hsa-miR-100-5p is associated with poor HCC prognosis [16,17,18,19], our research has discovered that its role in HCC prognosis in the Asian population is a unique finding that has caught our attention and prompted us to investigate it further.

hsa-miR-100-5p has been reported to inhibit several oncogenic genes that participate in various cancer-related signaling pathways [14,15]. To narrow down the list of potential miRNA targets, we used a database called miRTarBase, which contains experimentally validated miRNA-target interactions [33]. This systematic collection allowed us to compare the reliability of the interactions and ultimately led us to identify six targets. These targets are essential in various key cancer-related signaling pathways, including *PLK1*. Among these targets, *PLK1* is the only oncogene significantly impacting HCC prognosis. Several studies have suggested that low hsa-miR-100-5p expression is associated with *PLK1* overexpression in HCC [19,20]. Therefore, we decided to focus on investigating *PLK1* further.

Our study used a mouse liver cancer model based on HTVi to confirm the relationship between mir-100 and *PLK1* in vivo quickly. HTVi specifically transferred naked DNA plasmids into hepatocytes, resulting in a higher safety and incidence rate than conventional methods [36]. We also referenced recent studies and manipulated two oncogenes and two tumor suppressors, resulting in a rapidly generated primary HCC model [38,39,40]. This four-gene approach significantly reduced the modeling time and ensured more reliable results due to a more concentrated modeling cycle within four weeks. Using mouse liver cancer models, these results provide valuable insights for future rapid gene interaction studies and further validation of the relationship between hsa-miR-100-5p and *PLK1*.

PLK1 is a protein kinase crucial in regulating the cell cycle by phosphorylating various essential proteins during different cell stages [41]. In somatic cells, its expression is regulated by miRNA, increases during the S phase, and spikes during the G2 and M phases [42,43]. The overexpression of *PLK1*, found in various cancers, promotes cell proliferation, suppresses apoptosis, and is associated with poor prognosis due to its vital role in cell cycle regulation and vast pathway regulation [42,43]. Additionally, *PLK1* responds to DNA damage, which is associated with cancer drug resistance [44,45]. The high expression of *PLK1* has been identified as an independent risk factor for HCC in several studies [46,47,48]. PLK1 inhibition is already considered a potential target for cancer therapy, as it can lead to cancer cell death by interfering with multiple stages of mitosis [48,49]. Due to its miRNA-regulated nature and the importance of its role, *PLK1* is a suitable target for further investigation of hsa-miR-100-5p downregulation. Although several studies have analyzed the relationship between *PLK1* and HCC prognosis within public cohorts, there were hints that ethnic genetic variability may be involved, but it is still unclear how it affects it [50,51]. Our research revealed that both hsa-miR-100-5p and *PLK1* more significantly impact the survival of Asian patients with HCC. This led us to investigate the relationship between hsa-miR-100-5p and *PLK1*, and we discovered that the rs27770 variant is associated with the survival of Asian patients with HCC.

A previous genetic variability study of the *PLK1* gene demonstrated that the rs27770–G variant alters the *PLK1* mRNA secondary structure and remarkably reduces its mRNA stability [52]. We discovered that the frequency of rs27770–G was significantly higher in the Asian population. Bioinformatics analysis revealed that rs27770–G can not only alter PLK1 mRNA secondary structure but also lead to additional seedless miRNA binding sites that may further reduce *PLK1* mRNA stability under hsa-miR-100-5p suppression. These results offer insights into how genetic disparity due to race affects HCC prognosis by studying a variant of *PLK1*, a critical oncogene in HCC, in the Asian population.

Although we established that hsa-miR-100-5p is a specific prognostic marker for HCC in Asian patients, more systematic investigations and clinical experiments are still needed to give us a better understanding of the role of hsa-miR-100-5p in HCC pathogenesis. Bioinformatic prediction and population data strongly suggested that rs27770 of *PLK1* is associated with has-mir-100-5p as a specific prognostic marker in the Asian population. This miRNA-mRNA interaction must be validated in a larger sample.

## 5. Conclusions

This study confirmed that hsa-miR-100-5p is a tumor-suppressing miRNA targeting the well-known oncogene *PLK1*. hsa-miR-100-5p is commonly downregulated in HCC cell lines. Unusually low expression of hsa-miR-100-5p is associated with poor HCC prognosis, especially in the Asian population. *PLK1* expression is negatively correlated with hsa-miR-100-5p expression, resulting in significantly higher hazard rates in Asian patients with HCC. Moreover, SNP rs27770 located in the 3′UTR of *PLK1*, which has a higher G allele frequency in the Asian population, may render PLK1 mRNA unstable by altering its second structure and hsa-miR-100-5p binding capability, resulting in a remarkably negative effect of low hsa-miR-100-5p expression on HCC prognosis in the Asian population.

## Figures and Tables

**Table 1:** miRNA dysregulation in HCC cells that worsens HCC prognosis.

RNA Id	HepG2 *	Hep3B *	Huh7 *	Cox Coefficient **	*p*-Value **	Median Expression **	Mean Expression **
hsa-miR-100-5p	−7.323	−3.727	−7.086	−0.241	0.009	5468.529	7030.991
hsa-miR-769-5p	1.570	2.328	1.564	0.221	0.024	11.619	13.603
hsa-miR-1269a	3.897	6.359	7.028	0.188	0.048	102.494	703.047

* miRNA expression level compared to normal liver cells in log2(fold charge). ** Data acquired from OncoLnc database.

**Table 2:** List of hsa-miR-100-5p target genes with strong interaction evidence.

miRNA-mRNA Interaction			Strong Evidence *			Evidence Summary	
MIRT ID	miRNA	Target	Reporter Assay	Western Blot	q-PCR	Method Sum	Number of Publications
MIRT006429	hsa-miR-100-5p	IGF1R	Yes	Yes	Yes	4	4
MIRT000382	hsa-miR-100-5p	PLK1	Yes	Yes	Yes	4	6
MIRT003419	hsa-miR-100-5p	FGFR3	Yes	Yes	Yes	5	4
MIRT007365	hsa-miR-100-5p	MTOR	Yes	Yes	Yes	4	5
MIRT054544	hsa-miR-100-5p	HOXA1	Yes	Yes	Yes	5	2
MIRT054068	hsa-miR-100-5p	SMARCA5		Yes	Yes	3	5

* For each listed method, “Yes” represents the miRNA-mRNA interaction verified by this method in at least one publication.

**Table 3:** Different hsa-miR-100-5p expression groups in TCGA-LIHC data stratified by race, gender, and AJCC staging.

Patients’ Subgroups	Log-Rank Test Low mir-100-5p HR, 95% CI		Number of Cases (Number of Events in 5 Years)		Hazard Ratio (95% CI)	*p*-Value
			High mir-100-5p	Low mir-100-5p		
Race	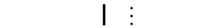					
American *	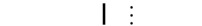		1(0)	0(0)	NA **	NA **
Asian	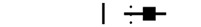		95(20)	42(24)	3.641 (1.794–5.853)	<0.0001
Black	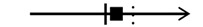		12(4)	3(2)	1.357 (0.1209–15.23)	0.7779
White	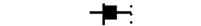		125(46)	31(17)	1.214 (0.6773–2.177)	0.4902
not reported	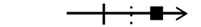		6(2)	2(2)	4.437 (0.3536–55.67)	0.0887
Gender	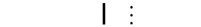					
female	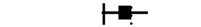		67(20)	33(20)	1.814 (0.9492–3.465)	0.0549
male	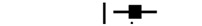		127(7)	45(24)	2.380 (1.294–4.379)	0.0003
AJCC staging	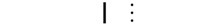					
T1 or T2	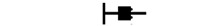		192(48)	47(20)	1.788 (0.9740–3.284)	0.0269
T3 or T4	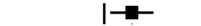		51(25)	33(24)	2.178 (1.186–3.998)	0.0045
All patients	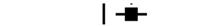		239(72)	78(44)	2.167 (1.397–3.363)	<0.0001
	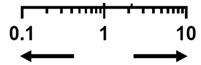					
	Favorable	Unfavorable				

* American race was short for American Indians and Alaska Natives. ** Cannot calculate due to insufficient cases in that particular subgroup.

## Data Availability

The datasets presented in this study can be found in online repositories. https://www.ncbi.nlm.nih.gov/geo/query/acc.cgi?acc=GSE215349 (accessed on 17 December 2023).

## Supplementary Materials

The following supporting information can be downloaded at: https://www.mdpi.com/article/10.3390/cancers16010129/s1, Supplementary Table S1: Primers used in this study. Supplementary Table S2: Densitometry readings of Western blots. Supplementary Table S3: Hardy–Weinberg equilibrium test for rs27770. Supplementary Table S4: Allele frequency of rs27770 from various human genetic variation projects. Supplementary Table S5: Full list of STarMir predict hsa-miR-100-5p binding sites in PLK1. Supplementary Figure S1: Uncropped Western blots. Supplementary Figure S2: Hematoxylin and Eosin (H&E) stained images of Mice HTVi Liver Cancer Model. Supplementary Data S1: Sanger sequencing data for rs27770 in the HCC cell lines.
