# Supplementary figures and images for: Increased hsa-miR-100-5p Expression Improves Hepatocellular Carcinoma Prognosis in the Asian Population with PLK1 Variant rs27770A>G
Source: Cancers (Basel). 2023 Dec 27;16(1):129. doi: 10.3390/cancers16010129 (PMC10778516; doi:10.3390/cancers16010129)

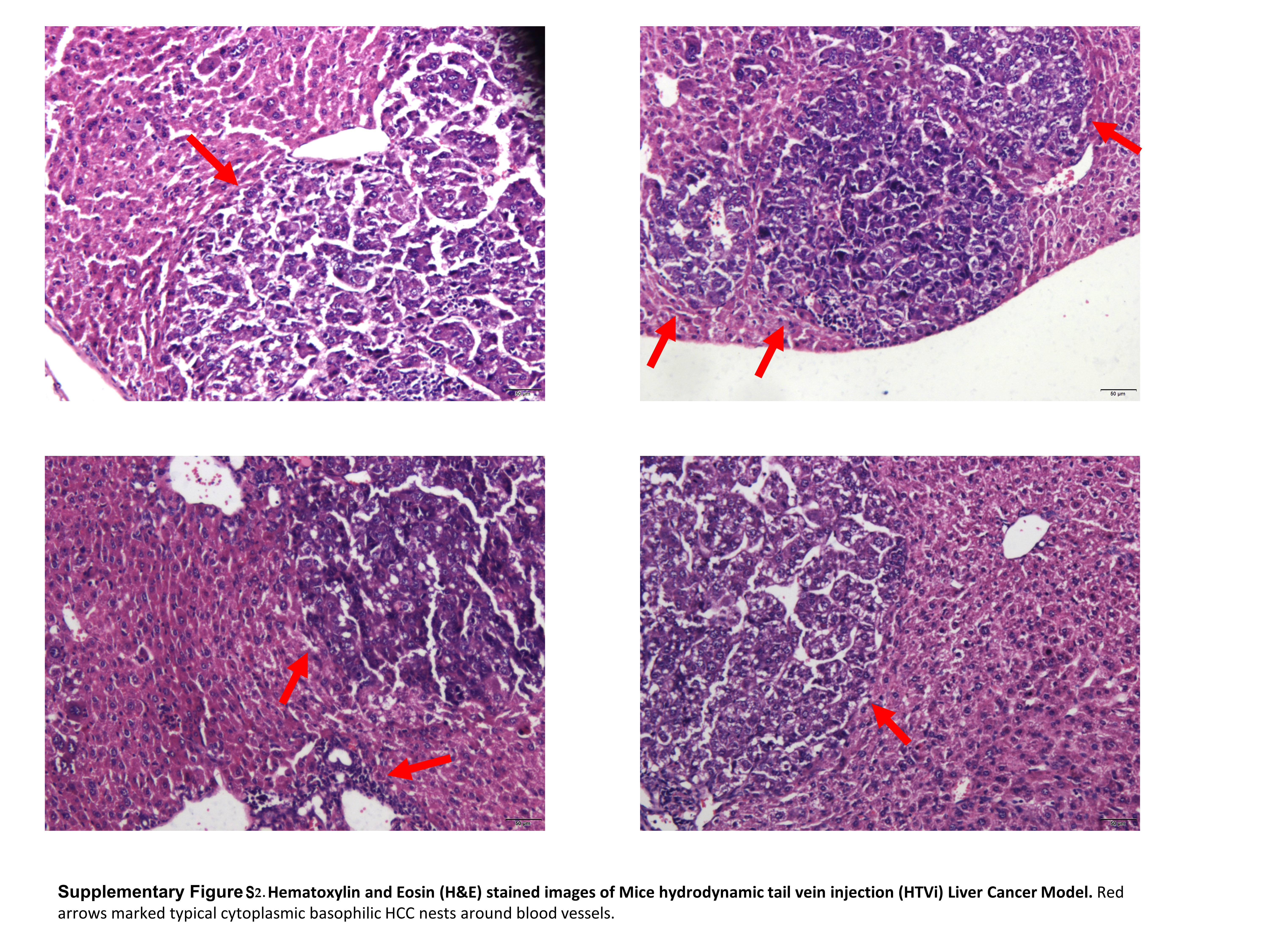

Supplement: Supplementary file 1 [file cancers-16-00129-s001.zip › Figure S2.tif]
