# Supplementary material for: Increased hsa-miR-100-5p Expression Improves Hepatocellular Carcinoma Prognosis in the Asian Population with PLK1 Variant rs27770A>G
Source: Cancers (Basel). 2023 Dec 27;16(1):129. doi: 10.3390/cancers16010129 (PMC10778516; doi:10.3390/cancers16010129)
Supplement: Supplementary file 1 [file cancers-16-00129-s001.zip › Data S1.zip!/aligment.pdf]

10 20 30 40 50 60 70 80 90 100 110 120 130 140 150

Start (0) BtgI MspA1I SacII BanI PstI BtsαI BaeGI Bsp1286I Bme1580I BglI AarI BfuAI BspMI PciI AflIII NspI NdeI

5′ CCCGCGGGTGCCATGTCTGCAGTGTGCCCCCCAGCCCCGGTGGCTGGGCAGAGCTGCATCATCCTTGCAGGTGGGGGTTGCTGTATAAGTTATTTTTGTACATGTTCGGGTGTGGGTTCTACAGCCT

3′ GGGCGCCCACGGTACAGACGTCACACGGGGGGTCGGGGCCACCGACCCGTCTCGACGTAGTAGGAACGTCCACCCCCAACGACATATTCAATAAAAACATGTACAAGCCCACACCCAAGATGTCGGA

CCCGCGGGTGCCATGTCTGCAGTGTGCCCCCCAGCCCCGGTGGCTGGGCAGAGCTGCATCATCCTTGCAGGTGGGGGTTGCTGTATAAGTTATTTTTGTACATGTTCGGGTGTGGGTTCTACAGCCTTGTCCCCCTCCCCCTCAACCCCACCATA

1 GCCATGTCTGCAGTGTGCCCCCCAGCCCCGGTGGCTGGGCAGAGCTGCATCATCCTTGCAGGTGGGGGTTGCTGTATAAGTTATTTTTGTACATGTTCGGGTGTGGGTTCTACAGCCTTGTCCCCCTCCCCCTCAACCCCACCATA

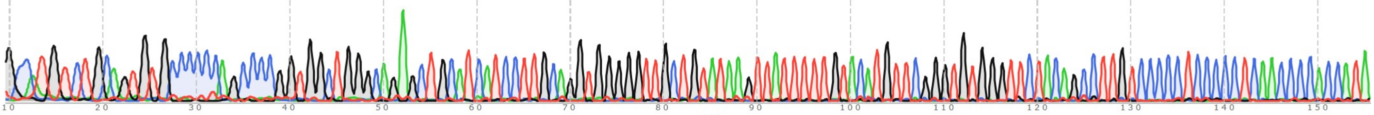

2 GG-TGCCATGTCTGCAGTGTGCCCCCCAGCCCCGGTGGCTGGGCAGAGCTGCATCATCCTTGCAGGTGGGGGTTGCTGTGTAAGTTATTTTTGTACATGTTCGGGTGTGGGTTCTACAGCCTTGTCCCCCTCCCCCTCAACCCCACCATA

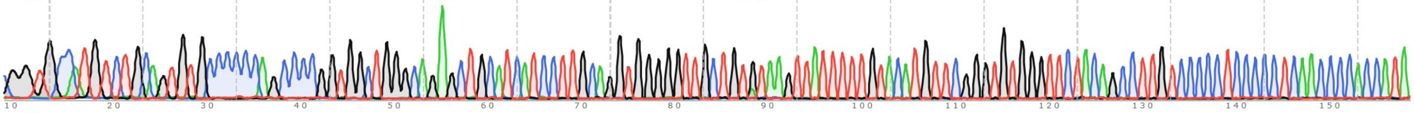

3 CGCGG-TGCCATGTCTGCAGTGTGCCCCCCAGCCCCGGTGGCTGGGCAGAGCTGCATCATCCTTGCAGGTGGGGGTTGCTGTATAAGTTATTTTTGTACATGTTCGGGTGTGGGTTCTACAGCCTTGTCCCCCTCCCCCTCAACCCCACCATA

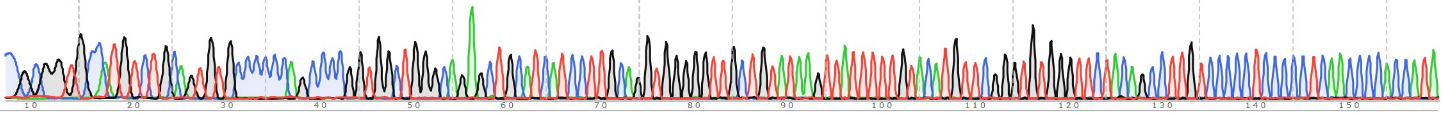

160 170 180 190 200 210 220

SspI ApoI EcoRI StyI *End* (220)

TGAATTGTACAGAATATTTCTATTGAATTCGGAACTGTCCTTTCCTTGGCTTTATGCACATTAAA

ACTTAACATGTCTTATAAAGATAACTTAAGCCTTGACAGGAAAGGAACCGAAATACGTGTAATTT

TGAATTGTACAGAATATTTCTATTGAATTCGGAACTGTCCTTTCCTTGGCTTTATGCACATTAAA

1 TGAATTGTACAGAATATTTCTATTGAATTCGGAACTGTCCTTTCCTTGGCTTTATGCACATTAAA

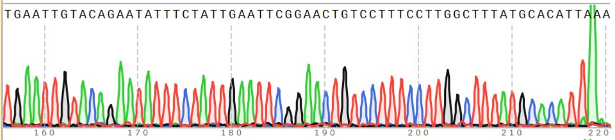

2 TGAATTGTACAGAATATTTCTATTGAATTCGGAACTGTCCTTTCCTTGGCTTTATGCACATTAAA

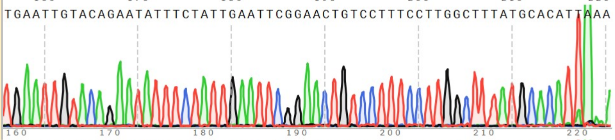

3 TGAATTGTACAGAATATTTCTATTGAATTCGGAACTGTCCTTTCCTTGGCTTTATGCACATTAAA

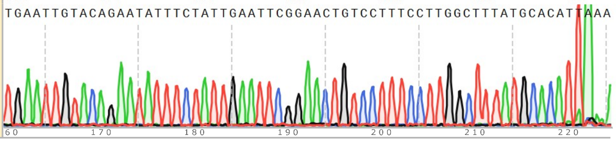

Original Sequence:

1: Huh7
220 bases
10 .. 220

2: HepG2
223 bases
10 .. 223 (1 mismatch, 1 gap)

3: Hep3B
224 bases
8 .. 224 (1 gap)
